# Analysis of the genotypic profile and its relationship with the clinical manifestations in people with cystic fibrosis: study from a rare disease registry

**DOI:** 10.1186/s13023-022-02373-y

**Published:** 2022-06-13

**Authors:** Senay Rueda-Nieto, Pedro Mondejar-Lopez, María-Pilar Mira-Escolano, Ana Cutillas-Tolín, Luis Alberto Maceda-Roldán, Julián Jesús Arense-Gonzalo, Joaquín A. Palomar-Rodríguez

**Affiliations:** 1 Teaching Unit of Preventive Medicine and Public Health, 30100 Murcia, Spain; 2 Paediatric Pulmonology and Cystic Fibrosis Unit, Virgen de La Arrixaca Clinic University Hospital (Murcia), 30120 Murcia, Spain; 3 Rare Diseases Information System, Planning and Health Financing Department, Regional Health Council, 30001 Murcia, Spain; 4 Division of Preventive Medicine and Public Health, Department of Public Health Sciences, University of Murcia School of Medicine, 30100 Murcia, Spain; 5 Institute for Biomedical Research of Murcia, IMIB-Arrixaca, 30120, El Palmar, Murcia, Spain

**Keywords:** Cystic fibrosis, CFTR, Genotype, Phenotype, Rare disease, Registry

## Abstract

**Background:**

Cystic fibrosis (CF) has a vast and heterogeneous mutational spectrum in Europe. This variability has also been described in Spain, and there are numerous studies linking CFTR variants with the symptoms of the disease. Most of the studies analysed determinate clinical manifestations or specific sequence variants in patients from clinical units. Others used registry data without addressing the genotype–phenotype relationship. Therefore, the objective of this study is to describe the genetic and clinical characteristics of people with CF and to analyse the relationship between both using data from the rare disease registry of a region in southeastern Spain.

**Methods:**

A cross-sectional study was carried out in people with a confirmed diagnosis of CF registered in the Rare Diseases Information System (SIER) of the Region of Murcia (Spain). The patients were classified into two genotypes according to the functional consequence that the genetic variants had on the CFTR protein.

**Results:**

There were 192 people diagnosed with CF reported in the Region of Murcia as of 31 December 2018. Seventy-six genotypes and 49 different variants were described, with c.1521_1523delCTT (p. Phe508del) being the most common in 58.3% of the CF patients and 37.0% of the alleles. In addition, 67% of the patients were classified as a high-risk genotype, which was associated with a lower percentage of FEV_1_ (OR: 5.3; 95% CI: 1.2, 24.4), an increased risk of colonization by *Pseudomonas aeruginosa* (OR: 7.5; 95% CI: 1.7, 33.0) and the presence of pancreatic insufficiency (OR: 28.1; 95% CI: 9.3, 84.4) compared to those with a low-risk genotype.

**Conclusions:**

This is the first study in Spain that describes the mutational spectrum and its association with clinical manifestations in patients with CF using data from a rare disease registry. The results obtained allow planning for the health resources needed by people with this disease, thus contributing to the development of personalized medicine that helps to optimize health care in CF patients.

**Supplementary Information:**

The online version contains supplementary material available at 10.1186/s13023-022-02373-y.

## Background

Cystic fibrosis (CF) (ORPHA: 586; OMIM: 219,700) is a rare genetic disease of autosomal recessive inheritance that is most common in the Caucasian population. [1]

This disorder originates in anomalies in the sequence of the CF transmembrane conductance regulator gene (CFTR) (OMIM 602,421), which cause an alteration in the chloride and bicarbonate transport channel regulated by cyclic adenosine monophosphate (cAMP). These alterations result in the appearance of various multisystemic clinical manifestations that generate a progressive deterioration in CF patients [2, 3].

Since the CFTR gene was first described in 1989 [4–6], 2107 variants have been reported in the cystic fibrosis mutation database [7], of which 431 are associated with the risk of disease [8]. The most common is c.1521_1523delCTT (p. Phe508del), which is present in more than 80% of alleles in the world population with CF and whose frequency is higher in northern European countries. In certain populations, other variants can reach higher frequencies than p. Phe508del, and some have only been described in specific territories [9–11].

Although p. Phe508del is also the most common CFTR gene variant in Spain, it is less common than in other northern European countries. Likewise, notable differences have been described between different regions of the country in the frequency of this sequence alteration, as well as great heterogeneity in other CFTR changes [12, 13].

The sequence variants are classified into 7 classes [14] according to the effect they have on the amount, function or stability of CFTR in the cell membrane [15, 16]. Recent studies have considered classifying sequence alterations into 2 groups: “minimal function variants” (I, II, and VII classes), which are considered high-risk mutations and are associated with a more severe phenotype and early deterioration, and “residual function variants”, or low-risk mutations (IV, V and VI classes), which can preserve some of the CFTR function and lead to milder late-onset disease [17, 18]. Class III variants (gating mutation) can belong to both classifications, although most are found among the minimal function variants [19]. However, it should be remembered that the variability or severity of CF symptoms also seems to be explained by factors such as age, disease progression, different environmental factors, and modifying genes [20, 21].

In addition, several studies have described an association between the genotype and different clinical manifestations, mainly reproductive, pancreatic and other gastrointestinal disorders, using different methods of classifying the genotype [22–25].

In Spain, studies have been carried out to analyse the genotype–phenotype relationship in patients from clinical units, which all include specific clinical manifestations or particular sequence variants [26, 27]. On the other hand, state-level studies have been carried out with data from registries that are not specific to CF [28, 29], in which a sample of CF patients was selected to describe certain characteristics without addressing the genotype–phenotype relationship.

Recently, rare disease registries have been positioned as a fundamental instrument since they allow greater knowledge of the epidemiology and characteristics of the people affected by obtaining systematic and complete information on each of them [30–32]. Therefore, the objective of this study was to describe the mutational spectrum as well as to analyse its relationship with the different clinical manifestations of people with CF based on the information from the rare disease registry of Murcia, a region of southeastern Spain.

## Methods

### Study population

A cross-sectional study was carried out among people with a confirmed diagnosis of CF through 31 December 2018, who were registered in the Rare Diseases Information System of Murcia (SIER) [33]. People with CFTR-related disorders (CFTR-RDs), CF-screen positive inconclusive diagnosis (CF-SPID) and healthy carriers were excluded. Informed consent of the study population was not needed, as the SIER is subject to personal data protection regulations and registered with the Spanish Data Protection Agency (no. 2101040243, on 14 April 2010) [34]. Even so, the study was presented to the Clinical Research Ethics Committee of the International Doctoral School of the University of Murcia (no. 3376/2021), and it was approved on 6 May 2021.

### Rare Diseases Information System (SIER)

The SIER, existing since 2010, is a population registry of rare diseases (RDR) of the Region of Murcia, an Autonomous Community located in southeastern Spain with an estimated population of 1,493,898 inhabitants as of 1 January 2019, which constitutes 3.18% of the Spanish population. For the inclusion of people with a rare disease (RD), this system uses a list of selected codes from the International Classification of Diseases (ICD) and integrates information from various sources. Currently, the SIER has 47 different sources of information: administrative clinics such as the regional Minimum Basic Data Set (MBDS); preexisting patient registries such as the renal disease registry; orphan or foreign drug dispensing database, and databases of people with recognition of disability and dependency; notifications from patient associations; and clinical hospital units. For this study, the Regional CF Unit of the Virgen de la Arrixaca University Clinic Hospital (HCUVA) was one of the main sources of information in the contribution of people with CF to the registry. Other sources that incorporated some of the study patients are shown in Table 1.

**Table 1:** Information sources that contribute CF patients to the SIER*

*Pre-existing records*
Regional Registry of the Minimum Basic Data Set (MBDS)
Database of People with Dependency in the Region of Murcia
Orphan drug dispensing registry of the Pharmaceutical Management Service^a^
Foreign drug dispensing registry^b^
Record of referral of patients to other Autonomous Communities
*Clinical units*
Center for Biochemistry and Clinical Genetics (CBGC)
HCUVA^c^ Medical Genetics Unit
Cystic Fibrosis Unit of de HCUVA^c^

*Each patient can be incorporated by more than one different source of information

^a^The orphan drug dispensing registry incorporated patients who had been dispensed with Cayston® and Kalydeco®

^b^The foreign drug dispensing registry incorporated patients who had been dispensed with Kemicetine® and Orkambi®

^c^HCUVA: Virgen de la Arrixaca Universitary Clinic Hospital

Once possible cases of RD have been incorporated into the registry, they undergo a validation process, confirming the evidence of the diagnosis once the electronic medical record of the patient has been reviewed [33].

Regarding the codes for the detection of people with CF, code 277.0 (0–9) from the International Classification of Diseases, Ninth Revision, Clinical Modification (ICD-9-CM) was used until 2015, and code E84 (0–9) from the tenth version of the Spanish Clinical Modification (ICD-10-ES) was used from 2016 to 2018, with no significant differences between the two classifications.

### Data collection

The data collected from each patient included the following:

*Consultation of basic patient information:* Sex, age at diagnosis (< 18 years or ≥ 18 years), age on 31 December 2018, native country of the parents, diagnosis by neonatal screening, death and transplant (yes/no).

*Obtaining genetic information*: First, information was obtained about the variants of the CFTR gene. In addition, the project database CFTR2 [8], the database of single nucleotide polymorphisms (dbSNP) [35], database of cystic fibrosis mutations [7] and ClinVAr [36] were consulted to include the type of alteration that patients presented in the gene sequence, along with the associated nucleotide change, amino acid change and its molecular and clinical consequence. Second, the patients were classified into 2 groups according to genotype: "high-risk" if the 2 alleles were made up of class I, II, III and VII variants (“minimal function mutations”) and "low-risk" if at least one allele carried a class IV, V, or VI variant (“residual function mutations”). A patient with the genotype "F508del/D1270N + R74W" was classified as low-risk because the complex allele was considered to improve the effect of the main mutation. In contrast, a patient with the genotype "G85E/G451V + G253R" was classified as high-risk because the complex allele was considered to worsen the effect of the parent mutation [1, 17, 18].

*Procurement of clinical manifestations:* We collected the following clinical manifestations through 31 December 2018: respiratory and digestive symptoms, metabolic disturbances and others such as bone alterations.

*Respiratory symptoms: *Evidence of at least one episode of allergic bronchopulmonary aspergillosis (ABPA), one or more clinically relevant episodes of haemoptysis (> 200 ml), the presence of nasal polyps, chronic respiratory colonization by different microorganisms (*Staphylococcus aureus*, *Burkholderia cepacia* or *Pseudomonas aeruginosa*) and at least one documented acute infection with methicillin-resistant *Staphylococcus aureus*, *Achromobacter xylosoxidans*, or nontuberculous mycobacteria.

Lung function was evaluated using the best value of the forced expiratory volume in the first second (FEV_1_) recorded in 2018, normalized with respect to its theoretical value using the Global Lung Function Initiative (GLI) tool and expressed as a percentage of the predicted value. The variable was dichotomized into ≤ 90% and > 90%, which is the cut-off point used in other studies [37].

*Digestive symptoms:* Presence of meconium ileus at birth, rectal prolapse, intussusception, distal intestinal obstruction syndrome (DIOS), pancreatic insufficiency, recurrent acute or chronic pancreatitis and CF-related liver disease (Liver disease with or without cirrhosis, including fatty liver).

*Metabolic disturbances:* Insulin-dependent CF-related diabetes (CFRD) and at least one CF-related episode of dehydration requiring medical attention.

*Others:* Bone disorders, including low bone density, osteoporosis, and digital arthropathy.

### Statistical analysis

We described the clinical and demographic variables in the two groups of genotypes established by a hypothesis contrast test according to the type of variables and their normality. The normality test was carried out using the Kolmogorov–Smirnov test. The absolute and relative frequencies of the clinical and demographic variables were evaluated. The allelic frequencies of the CFTR gene variants in the studied population were also evaluated.

For the quantitative variables, Student's t test was used if the data were normally distributed, and the Mann–Whitney U test was used if they were not. For qualitative variables, the chi-squared or Fisher’s exact test was used when applicable.

Additionally, crude and adjusted odds ratio (OR) and 95% confidence interval (CI) were calculated using binary logistic regression analysis to examine associations between genotype and the clinical manifestations of the participants. There was a statistically significant association between genotype and age at diagnosis and age as of 31 December 2018 (*p* < 0.01). Therefore, these variables were taken into account for the adjustment of the model together with sex and native country of the parents.

In addition, a sensitivity analysis was performed to verify that the patients diagnosed by neonatal screening did not lead to bias.

All tests were two-tailed, and the level of statistical significance was established at ≤ 0.05. Statistical analyses were performed with the IBM SPSS 25.0 statistical package (IBM Corporation, Armonk, New York, USA).

## Results

There were 192 people diagnosed with CF registered in the SIER through 31 December 2018.

Of the total number of people included in the study, 53.6% were male, with a mean age ± standard deviation (SD) of 20.0 ± 15.2 years (median: 15.0, interquartile range [IQR]: 7.0–31.0), and 46.4% were female (mean ± SD: 24.5 ± 16.2 years, median: 23.0, IQR: 10.0–35.0). Adults (18 years or older) comprised 41.7% of all patients. The mean age ± SD at diagnosis was 7.8 ± 14.4 years, and the median was 0.0 years (IQR 0.0–7.5). Moreover, 16.1% of people (n = 31) were diagnosed by the neonatal screening program, which was implemented in Murcia in March 2007.

In 84.9% of the study population, the native country of the parents was Spain. In descending order of frequency, parents had other nationalities as follows: Ecuadorian (6.2%), English (2.6%), Moroccan (2.1%), and Argentine (1.0%). The remaining 3.2% included parents of French, Peruvian, Moldovan, Ukrainian, Hungarian and Bulgarian nationalities.

As a result of clinical manifestations, respiratory problems were present in 63.0% of the patients. The mean FEV_1_ percentage ± SD was 90.0 ± 21.4 and was inversely correlated with the age of the patients (−0.36; *p* < 0.01). In addition, 57.8% of the people presented infection/colonization by a bacterial pathogen at some point. The most frequently isolated microorganism was *Staphylococcus aureus*, with those under 18 years of age being the most likely to be infected. Among digestive manifestations, pancreatic insufficiency was the most common (56.8%). Furthermore, 8.9% of the patients presented meconium ileus as the first manifestation of the disease.

Table 2 shows the main demographic and clinical characteristics of the patients according to their genotype, available for 94.8% of the cases (n = 182). Patients for whom genetic information was not available (n = 10) were excluded from further statistical analyses. Of these patients, 67% were classified as having a high-risk genotype (n = 122), and 33% were classified as having a low-risk genotype (n = 60).

**Table 2:** Demographic and clinical characteristics according to genotype in patients with cystic fibrosis*

	*High risk* Genotype	*Low risk* Genotype	Total (*n* = 182)	*p*-value (*p* ≤ 0.05)
	*No. patients/No. studied (%)*			
*Demographics characteristics*				
Male sex	64/122 (52.5)	35/60 (58.3)	99/182 (54.4)	0.454
Age (years)^a^. Median (25–75)^b^	15.0 (7.0–28.0)	28.5 (11.8–42.0)	19.0 (8.0–33.0)	< 0.001
Age at diagnosis < 18 years	114/117 (97.4)	40/60 (66.7)	154/177 (87.0)	< 0.001
Death	30/122 (24.6)	2/60 (3.3)	32/182 (17.6)	< 0.001
Lung or liver transplant	19/122 (15.6)	4/60 (6.7)	23/182 (12.6)	0.089
*Clinical characteristics*				
*Respiratory manifestations*				
FEV_1_ as % predicted^c^. Mean ± SD^d^	87.1 ± 20.5 (57)	94.1 ± 22.2 (40)	90.0 ± 21.4 (97)	0.045
Nasal polyposis	18/87 (20.7)	14/51 (27.5)	32/138 (23.2)	0.364
Haemoptysis	18/87 (20.7)	12/51 (23.5)	30/138 (21.7)	0.696
ABPA^e^	11/87 (12.5)	4/51 (7.8)	15/138 (10.9)	0.572
Infection by^*f*^				
*Staphylococcus aureus*	55/87 (63.2)	24/50 (48.0)	79/137 (57.7)	0.044
MRSA	10/87 (11.5)	0/50 (0.0)	10/137 (7.3)	0.013
*Pseudomonas aeruginosa*	31/88 (35.2)	12/51 (23.5)	43/139 (30.9)	0.150
*Achromobacter xylosoxidans*	14/87 (16.1)	2/50 (4.0)	16/137 (11.7)	0.034
*Burkholderia cepacia*	1/87 (1.1)	1/50 (2.0)	2/137 (1.5)	0.689
Non-tuberculous *mycobacteria*	6/87 (7.1)	3/50 (6.0)	9/137 (6.6)	0.838
*Gastrointestinal manifestations*				
Meconium ileus	17/102 (16.7)	0/59 (0.0)	17/161 (10.6)	0.001
Pancreatic insufficiency	91/102 (89.2)	18/59 (30.5)	109/161 (67.7)	< 0.001
Pancreatitis^g^	4/87 (4.6)	4/51 (7.8)	8/138 (5.8)	0.467
Liver disease^h^	15/89 (16.9)	1/52 (1.9)	16/141 (11.3)	0.007
Rectal prolapse	4/87 (4.6)	0/51 (0.0)	4/138 (2.9)	0.296
Intussusception	3/88 (3.4)	0/51 (0.0)	3/139 (2.2)	0.298
DIOS^i^	8/87 (9.2)	2/51 (3.9)	10/138 (7.2)	0.323
*Metabolic disturbances*				
CF-related diabetes	14/88 (15.9)	1/52 (1.9)	15/140 (10.7)	0.010
Clinically significant dehydration	14/87 (16.1)	9/51 (17.6)	23/138 (16.7)	0.817
Bone alterations^j^	11/87 (12.6)	4/52 (7.7)	15/139 (10.8)	0.413

*Manifestations that have been present at some point in the patient's life through December 31, 2018. The genotype information of ten patients is unknown

^a^Age on December 31st, 2018

^b^25-75 = 25th–75th percentile

^c^Forced Expiratory Volume in the first second (Percentage of predicted value). The best value of the year 2018 was measured

^d^SD = Standard deviation

^e^ABPA = Allergic Bronchopulmonary Aspergillosis

^f^It includes chronic colonization by *Staphylococcus aureus*, *Pseudomonas aeruginosa* and *Burkholderia cepacia*, and some acute infection by methicillin-resistant *Staphylococcus aureus* (MRSA), *Achromobacter xylosoxidans* and non-tuberculous *mycobacteria*

^g^Recurrent acute or chronic pancreatitis

^h^Cirrhosis or liver disease without cirrhosis, including fatty liver

^i^DIOS = Distal Intestinal Obstruction Syndrome

^j^It includes low bone density, osteoporosis and a digital arthropathy

People with a high-risk genotype were younger (*p* < 0.001), with a lower mean age at diagnosis (*p* < 0.001) and lower mean FEV_1_ values (*p* = 0.045) with respect to the low-risk genotype. Likewise, the high-risk genotype presented a higher frequency of respiratory infections by methicillin-resistant *Staphylococcus aureus* (*p* = 0.013) and *Achromobacter xylosoxidans* (*p* = 0.034). A higher incidence of meconium ileus, pancreatic insufficiency, CF-related liver disease and CFRD was observed in the high-risk patients (*p* ≤ 0.01). Furthermore, 15.6% of patients with a high-risk genotype required lung or liver transplantation compared to 6.7% with a low-risk genotype, although the difference was not significant (*p* = 0.089).

Table 3 shows the frequency of CFTR gene variants by alleles in the 192 patients studied. The most common mutation was p. Phe508del in 58.3% of the patients (27.0% homozygous and 73.0% heterozygous) and 37.0% of the alleles.

**Table 3:** CFTR sequence variants detected in 384 alleles from 192 patients studied

CFTR variant (Classic nomenclature)	Nucleotide/protein (Standard nomenclatura)	Variant typeª	Molecular consequence	Clinical significance	Frequency of alleles. No. (%)	Effect on CFTR^b,c^
F508del	c.1521_1523delCTT/ p.Phe508del	Deletion	Inframe deletion	Pathogenic	142 (37.0)	MF
G542X	c.1624G > T/p.Gly542Ter	SNV	Nonsense	Pathogenic	31 (8.1)	MF
A1006E	c.3017C > A/p.Ala1006Glu	SNV	Missense	Pathogenic	17 (4.4)	RF
L206W	c.617 T > G/p.Leu206Trp	SNV	Missense	Pathogenic	15 (3.9)	RF
2789 + 5G > A	c.2657 + 5G > A/*	SNV	Splicing	Pathogenic	13 (3.4)	RF
K710X	c.2128A > T/p.Lys710Ter	SNV	Nonsense	Pathogenic	13 (3.4)	MF
H609R	c.1826A > G/p.His609Arg	SNV	Missense	Pathogenic	12 (3.1)	MF
1811 + 1.6kbA > G	c.1680-886A > G/*	SNV	Splicing	Pathogenic	10 (2.6)	MF
R334W	c.1000C > T/p.Arg334Trp	SNV	Missense	Pathogenic	10 (2.6)	RF
N1303K	c.3909C > G/p.Asn1303Lys	SNV	Missense	Pathogenic	9 (2.3)	MF
G85E	c.254G > A/p.Gly85Glu	SNV	Missense	Pathogenic	8 (2.1)	MF
2869insG	c.2737_2738insG/p.Tyr913Ter	Insertion	Nonsense	Pathogenic	7 (1.8)	MF
3849 + 10kbC > T	c.3718-2477C > T/*	SNV	Splicing	Pathogenic	6 (1.6)	RF
711 + 1G > T	c.579 + 1G > T/*	SNV	Splice donor	Pathogenic	6 (1.6)	MF
I507del	c.1516ATC[1]/p.Ile507del	Microsatellite	Inframe deletion	Pathogenic	6 (1.6)	MF
R347P	c.1040G > C/p.Arg347Pro	SNV	Missense	Pathogenic	6 (1.6)	MF
R560G	c.1678A > G/p.Arg560Gly	SNV	Missense	Not provided	4 (1.0)	MF
D1152H	c.3454G > C/p.Asp1152His	SNV	Missense	Pathogenic	3 (0.8)	RF
5 T-TG12	c.[1210-34TG[12];1210–12 T[5]]/*	Deletion	Intron variant	Conflicting interpretations of pathogenicity	3 (0.8)	RF
2183AA > G	c.2051_2052delinsG/ p.Lys684fs	Indel	Frameshift	Pathogenic	2 (0.5)	MF
A561E	c.1682C > A/p.Ala561Glu	SNV	Missense	Pathogenic	2 (0.5)	MF
CFTRdele22,23	c.3964-78_4242 + 577del/*	Deletion	Splice acceptor splice donor	Pathogenic	2 (0.5)	MF
L1254X	c.3761 T > G/p.Leu1254Ter	SNV	Nonsense	Pathogenic	2 (0.5)	MF
Q890X	c.2668C > T/p.Gln890Ter	SNV	Nonsense	Pathogenic	2 (0.5)	MF
R1162X	c.3484C > T/p.Arg1162Ter	SNV	Nonsense	Pathogenic	2 (0.5)	MF
S549R	c.1647 T > G/p.Ser549Arg	SNV	Missense	Pathogenic	2 (0.5)	MF
1609delCA	c.1477_1478del/p.Gln493fs	Deletion	Frameshift	Pathogenic	1 (0.3)	MF
1716G > A	c.1584G > A/p.Glu528 =	SNV	Synonymous	Conflicting interpretations of pathogenicity	1 (0.3)	MF
1717-1G > A	c.1585-1G > A/*	SNV	Splice acceptor	Pathogenic	1 (0.3)	MF
1898 + 1G > A	c.1766 + 1G > A/*	SNV	Splice donor	Pathogenic	1 (0.3)	MF
2603delT	c.2472del/p.Asn825fs	Deletion	Frameshift	Pathogenic	1 (0.3)	MF
3195del6	c.3067_3072del/ p.Ile1023_Val1024del	Deletion	Inframe Deletion	Pathogenic/Likely pathogenic	1 (0.3)	MF
3849 + 1G > A	c.3717G > A/p.Arg1239 =	SNV	synonymous	Pathogenic	1 (0.3)	RF
621 + 1G > T	c.489 + 1G > T/*	SNV	Splice donor	Pathogenic	1 (0.3)	MF
712-1G > T	c.580-1G > T/*	SNV	Splice acceptor	Pathogenic	1 (0.3)	MF
A534E	c.1601C > A/p.Ala534Glu	SNV	Missense	Uncertain significance	1 (0.3)	RF
D1270N + R74W**	c. [220C > T; 3808G > A]. c.220C > T/ (p.Arg74Trp)	Haplotype	No data	Uncertain significance​	1 (0.3)	RF
E1308X	c.3922G > T/p.Glu1308Ter	SNV	Nonsense	Likely pathogenic	1 (0.3)	MF
E585X	c.1753G > A/p.Glu585Ter	SNV	Nonsense	Pathogenic	1 (0.3)	MF
G451V + G253R**	c.1352G > T/p.Gly451Val. c.757G > A/p.Gly253Arg	Haplotype	No data	Uncertain significance	1 (0.3)	RF
G85V	c.254G > T/p.Gly85Val	SNV	Missense	Pathogenic	1 (0.3)	MF
L15P	c.44 T > C/p.Leu15Pro	SNV	Missense	Pathogenic	1 (0.3)	MF
R1066C	c.3196C > T/p.Arg1066Cys	SNV	Missense	Pathogenic	1 (0.3)	MF
R1158X	c.3472C > T/p.Arg1158Ter	SNV	Nonsense	Pathogenic	1 (0.3)	MF
R117H	c.350G > A/p.Arg117His	SNV	Missense	Pathogenic	1 (0.3)	RF
V562I	c.1684G > C/p.Val562Ile	SNV	Missense	Conflicting interpretations of pathogenicity	1 (0.3)	RF
W1089X	c.3266G > A/p.Trp1089Ter	SNV	Nonsense	Pathogenic	1 (0.3)	MF
W1282X	c.3846G > A/p.Trp1282Ter	SNV	Nonsense	Pathogenic	1 (0.3)	MF
W202X	c.606G > A, p.Trp202Ter	SNV	Nonsense	Not provided	1 (0.3)	MF
Unknown data	–	–	–	–	25 (6.5)	–

*No protein name

**Complex alleles

^a^SNV: Single nucleotide variant

^b^MF: Minimal function mutation

^c^RF: Residual function mutation

In total, 76 genotypes and 49 different variants were found. In approximately 50% of the alleles, the following 3 mutations were observed: p. Phe508del, c.1624G > T (p.Gly542Ter) and c.3017C > A (p.Ala1006Glu). Other variants were found in 1.6% to 3.9% of the alleles, generally in compound heterozygosity with other “residual function mutations” or with p.Phe508del. The rest of the variants were presented in frequencies equal to or less than 1%.

Table 4 shows the multivariate analysis of the relationship between genotype and clinical manifestations. The high-risk genotype was significantly associated with a lower percentage of FEV_1_ values (OR: 5.3; 95% CI: 1.2, 24.4), a higher risk of developing *Pseudomonas aeruginosa* infection (OR: 7.5; 95% CI: 1.7, 33.0) and the presence of pancreatic insufficiency (OR: 28.1; 95% CI: 9.3, 84.4) (*P* < 0.05) compared to the low-risk genotype. No other statistically significant associations were observed.

**Table 4:** Multivariate analysis for the clinical manifestations of people with cystic fibrosis and their genotype*

Variables	Genotype					
	Unadjusted			Adjusted		
	OR	95% CI	*p*-value	OR	95% CI	*p*-value
%FEV_1_**	1.8	0.73–3.84	0.226	5.3	1.15–24.41	0.032
*S. Aureus* infection	1.9	0.92–3.77	0.084	1.8	0.76–4.44	0.174
MRSA infection	3.2	0.70–14.86	0.136	2.6	0.35–18.97	0.350
*Pseudomonas aeruginosa* infection	1.8	0.81–3.84	0.155	7.5	1.72–33.00	0.007
*Achromobacter xylosoxidans* infection	4.5	0.99–20.87	0.052	5.1	0.72–35.42	0.103
Meconium ileus	4.7	1.19–18.64	0.028	3.2	0.71–14.50	0.132
Pancreatic insufficiency	17.2	7.57–39.01	< 0.001	28.1	9.33–84.44	< 0.001
Liver disease	3.7	0.98–14.30	0.053	3.9	0.72–20.61	0.115
CF-related diabetes	3.5	3.52–13.51	0.067	3.4	0.78–15.12	0.102

Adjusted model controlled by age at diagnosis and through December 31 2018, sex and native country of the parents

*Results for high-risk genotype using low risk genotype as a reference

**Dichotomized in ≤ 90% and > 90%

## Discussion

The present study shows CFTR sequence alterations and their relationship with the clinical manifestations of people with CF included in the rare disease registry of the Region of Murcia. Although the mutational spectrum of CFTR in Murcia was published in 2009 [38], the study included 91 patients selected from the CF unit in whom 29 different variants were described. Therefore, our study offers more complete, up-to-date and representative information on the genetics of people with CF in this geographic area. Furthermore, we have no evidence of other regional or national articles that analyse the genotype–phenotype relationship among patients included in this type of registry.

In the study population, the most frequently observed variants were p. Phe508del, p.Gly542Ter. and p.Ala1006Glu. Additionally, 76 genotypes and 49 different variants were detected, supporting the great heterogeneity described in Mediterranean countries [9, 39].

Phe508del is the most common sequence change in 58.3% of CF patients, a figure lower than that reported by other Spanish Autonomous Communities [40, 41] and different European Mediterranean countries [1, 11, 42–44]. In addition, its frequency by alleles is among the lowest data described to date (37.0%) due in large part to the high percentage of carriers of the variant in heterozygosity (73%) compared with the 48.1% reported recently by the registry of CF patients in Spain [13].

Moreover, the Region of Murcia constitutes, together with Andalusia and the Balearic Islands, as one of the Spanish Autonomous Communities with the highest percentage of alleles with the variant p.Gly542Ter [41, 45]. In fact, according to a study by Estivill et al. [12], this alteration is more common in Mediterranean countries, with an average frequency of 6.1%, and the highest prevalence described thus far was in the Balearic Islands (16.7%). Recent data from the Spanish CF registry suggest that 7.7% of registered patients carry the p.Gly542Ter variant [13]. In the SIER, this variant is present in 16.5% of patients and 8.1% of CFTR alleles.

The variants described above were followed in frequency by p. Ala1006Glu and c.617 T > G (p. Leu206Trp), which is rare in the rest of European countries [11, 13]. Nevertheless, other frequent mutations in Europe, such as c.1652G > A (p. Gly551Asp), which have a specific treatment [46], have not been described in our study population.

The patients were grouped into 2 genotypes according to the consequence that the different variants have on the function and amount of CFTR protein, as proposed by previous studies^,^ [47]. However, to date, no work has used this classification to link the genotype to all the clinical manifestations included in this study.

The proportion of people classified as having the low-risk genotype was 33%. This represents a much higher percentage of patients with mild forms than that described by McKone [17] or De Boeck [47] in other countries, while studies in Mediterranean countries indicate that this figure is close to 15% [48]. In Spain, there are no studies that describe the percentage of mild forms, but the figure reported in this study supports what has been suggested by other authors such as De Gracia, who points out that the mild forms could be more frequent than what has been described to date [49].

The frequency of a high percentage of people with a low-risk genotype can largely explain the presence of the different clinical manifestations. An example of this is pancreatic insufficiency, which existed in 67.7% of the patients in this study. Although it has been classically described that approximately 85–90% of CF cases are associated with pancreatic insufficiency, our results show that this percentage may be compatible with severe forms, since pancreatic insufficiency was present in 89.2% of our cases classified as high-risk versus 30.5% classified as low-risk.

In addition, our results are consistent with previous studies that have described an association between genotype and respiratory and digestive symptoms in people with CF. In our study population, the most consistent findings observed were the appearance of pancreatic insufficiency with the high-risk genotype but also a lower percentage of predicted FEV_1_ and colonization by *Pseudomonas aeruginosa*.

The relationship between the severity of the mutations and pancreatic damage has been previously reported by grouping the mutations into classes, associating a higher risk of pancreatic insufficiency in those with variants that cause greater CFTR dysfunction [50–53].

Regarding colonization by microorganisms, Kerem et al. [54] and Vongthilath et al. [55] showed that chronic infection by *Pseudomonas aeruginosa* tends to appear in CF populations that present greater lung damage and more severe symptoms related to loss of function of CFTR. Therefore, although different authors have linked the genotype with pancreatic function and lung damage, to date, no studies have used this classification. So, we consider that the grouping used allows the entire mutational spectrum to be combined into only two well-differentiated groups (high and low risk), constituting a more useful way of approaching these studies and of knowing the prognosis of the disease in a simpler way.

Various studies also described a relationship between genetics and infection by other microorganisms [56]; however, we did not find statistically significant associations in this regard. The same occurred with complications such as CFRD and CF-related liver disease, in which no significant association was observed in the adjusted model.

Considering the limitations of the study, the relatively small population studied could make it difficult to detect potential effects. However, in our study statistically significant associations were found for different manifestations. Even so, we cannot rule out the appearance of a type II error for the variables in which no statistically significant differences were found.

Although not all clinical information was available for all patients, there were no significant differences between participants with or without information about genotype or age, so information bias is unlikely. Additionally, although this study analysed a large set of clinical manifestations and related diseases, there are some that were not addressed, such as nutritional status, infertility or oncological pathology, which may be included in future work.

The possibility that the neonatal screening diagnosis could act as a modifier of the association between clinical manifestations and genotype could be considered a limitation of our study. Nevertheless, we carried out a sensitivity analysis and verified that the associations were similar when analysing both groups separately, so it was concluded that there was no modification of the effect by screening.

Furthermore, it should be noted that not all the people with CF in the study had the same time of evolution of the disease. In fact, certain conditions, such as pancreatic insufficiency, are described as being closely related to age. Notwithstanding, a significant association was obtained for this relationship in the studied population when we adjusted the model by age and age at diagnosis.

It is worth mentioning that one of the main strengths of our study is the use of a population-based registry, which offers up-to-date and extensive information on these patients and allows us to know the frequency, distribution, evolution and needs of the patients affected by CF or other rare diseases. In addition, the SIER offers representative data of those affected by the disease, since it is estimated that it has registered all of these patients due to the high number of sources and the obligation of them in sending their information, constituting the reference registry for regional data [34]. For all these reasons, knowing their characteristics and their mutational spectrum, we can determine which patients would benefit from new treatments, such as highly effective CFTR-modulating therapies, where currently 16% of the people studied had received any such treatment.

However, future studies are needed to address these aspects, taking into account the changes in the eligibility criteria of some of the treatments, the incorporation of therapies recently approved in Spain, such as the combination of tezacaftor, ivacaftor and elexacaftor, and the impact that these treatments have on the progression of CF. In addition, new studies carried out using other Spanish regional registries or in a broader population such as that of the state RD registry with the methodology proposed here, could provide more information and support the results obtained [57].

## Conclusions

To our knowledge, this is the first Spanish study to describe the mutational spectrum and its association with clinical manifestations in people with CF included in a rare disease registry. The frequency of the p. Phe508del variant was one of the lowest described in Europe, and the percentage of people classified as having a low-risk genotype was higher than that described by other authors.

In addition, the high-risk genotype increased the risk of severe lung damage, pancreatic insufficiency and chronic respiratory colonization by *Pseudomonas aeruginosa* in comparison with the low-risk genotype.

The results obtained in this work allow for planning of the resources that health services must provide to people with CF, contributing to the development of public health strategies to move towards personalized precision medicine that helps to optimize the health care for these patients.

## Supplementary Information

**Additional file 1**.** Table S1**. Classification of genotypes according to risk.

## Data Availability

The pseudoanonymised dataset used to carry out this study and to support its findings are restricted following Regulation (EU) 2016/679, Law 3/2018 on the Protection of Personal Data, Law 14/2007 on Biomedical Research, and Laws 37/2007 and 18/2015 on the Reuse of Public Sector Information. Based on the foregoing, it is only possible to access the aggregated data with a reasonable request at the following address: serplan@listas.carm.es.

## Abbreviations

cAMP: Cyclic adenosine monophosphate
CF: Cystic fibrosis
CFRD: Cystic fibrosis-related diabetes mellitus
CF-SPID: Cystic fibrosis screen positive inconclusive diagnosis
CFTR: Cystic fibrosis transmembrane conductance regulator
CFTR-RDs: Cystic fibrosis transmembrane conductance regulator-related disorders
CI: Confidence interval
FEV1: Forced expiratory volume in the first second
HCUVA: Virgen de la Arrixaca University Clinic Hospital
ICD: International classification of diseases
IQR: Interquartile range
MRSA: Methicillin-resistant *Staphylococcus aureus*
OR: Odds ratio
PI: Pancreatic insufficiency
PS: Pancreatic sufficiency
RD: Rare disease
RDR: Rare disease registry
SIER: Rare Diseases Information System
