# Supplementary material for: Analysis of the genotypic profile and its relationship with the clinical manifestations in people with cystic fibrosis: study from a rare disease registry
Source: Orphanet J Rare Dis. 2022 Jun 13;17:222. doi: 10.1186/s13023-022-02373-y (PMC9195274; doi:10.1186/s13023-022-02373-y)
Supplement: Supplementary file 1 — Additional file 1. Table S1. Classification of genotypes according to risk. [file 13023_2022_2373_MOESM1_ESM.docx]

| **Additional File 1: Table S1 Classification of genotypes according to risk** | | |
| --- | --- | --- |
| **Risk** | **Variants function** | **Genotypes** |
| High-risk | Minimal function  +  Minimal function | 1609delCA/CFTRdele22,23. 2183AA>G/1677delTA. 712-1G>T/2869insG. 711+1G>T/1811+1,6kbA>G. A561E/A561E  CFTRdele22,23/711+1G>T. F508del/1898+1G>A. F508del/1811+1,6kbA>G. F508del/2183AA>G. F508del/2869insG. F508del/2603delT. F508del/621+1G>T.  F508del/711+1G>T. F508del/F508del. F508del/G542X. F508del/G85E. F508del/I507del. F508del/K710X. F508del/Other unidentified. F508del/L15P. F508del/L1254X. F508del/N1303K.  F508del/R347P. F508del/W1282X. F508del/S549R. F508del/W202X. G542X/Other unidentified. G542X/G85E.  G542X/N1303K. G542X/G542X. G85V/R1066C.  G85E/G451V+G253R. G85E/G85E. H609R/H609R. I507del/Other unidentified. K710X/G542X  K710X/1811+1,6kbA>G. K710X/K710X. N1303K/2869insG  K710X/R560G. 1716G>A/Other unidentified.  R1162X/K710X. R1162X/G542X. R347P/R1158X. R347P/K710X. S549R/G85E. |
| Low risk | Minimal function  +  Residual function | 2789+5G>A/1811+1,6kbA>G. 1717-1G>A/2789+5G>A.  A1006E/2869insG. A1006E/K710X.  A534E/W1089X. D1152H/1811+1,6kbA>G. E585X/A1006E.  F508del/2789+5G>A. F508del/3849+10kbC>T. F508del/5T-TG12. F508del/A1006E. F508del/D1152H. G542X/A1006E  F508del/D1270N+R74W. F508del/L206W. I507del/L206W.  G542X/3849+10kbC>T. L206W/1811+1,6kbA>G.  L206W/3195del6. L206W/R347P. N1303K/L206W.  N1303K/A1006E. Q890X/L206W. Q890X/R117H.  R560G/2789+5G>A. E1308X/R334W. F508del/R334W  G542X/R334W. R334W/R347P. |
|  | Residual function  +  Residual function | 2789+5G>A/A1006E. A1006E/R334W.  L206W/3849+1G>A. R334W/L206W. V562I/5T-TG12 |
